# Evaluating the perceived added value of a threefold intervention to improve palliative care for persons experiencing homelessness: a mixed-method study among social service and palliative care professionals

**DOI:** 10.1186/s12904-022-01000-8

**Published:** 2022-06-23

**Authors:** Hanna T. Klop, Anke J. E. de Veer, Jaap R. G. Gootjes, Dike van de Mheen, Igor R. van Laere, Marcel T. Slockers, Bregje D. Onwuteaka-Philipsen

**Affiliations:** 1 Department of Public and Occupational Health, Amsterdam UMC, Vrije Universiteit Amsterdam, Amsterdam Public Health research institute (APH), Van der Boechorststraat 7, 1081BT Amsterdam, Netherlands; 2 Netherlands Institute for Health Services Research (Nivel), Utrecht, Netherlands; 3 Hospice Kuria, Amsterdam, Netherlands; 4 Expertise Centre for Palliative Care, Amsterdam UMC location VUmc, Amsterdam, Netherlands; 5 Tranzo Scientific Center for Care and Wellbeing, Tilburg University, Tilburg, Netherlands; 6 Netherlands Street Doctors Group, Rotterdam, Netherlands; 7 Centrum voor Diensten (CVD) Havenzicht, Rotterdam, Netherlands

**Keywords:** Palliative care, Homeless, End of life, Health care, Social services, Intervention

## Abstract

**Background:**

Palliative care for persons experiencing homelessness who reside in social service facilities is often late or lacking. A threefold intervention was implemented to improve palliative care for this population by increasing knowledge and collaboration between social service and palliative care professionals. This consultation service comprised: 1) consultations between social service professionals and palliative care professionals; 2) multidisciplinary meetings involving these professionals; and 3) training of these professionals. This study aims to evaluate the perceived added value of this threefold consultation service in three regions in the Netherlands.

**Methods:**

A mixed-methods evaluation study using structured questionnaires for consultants, requesting consultants, and attendees of multidisciplinary meetings, semi-structured group and individual interviews with social service and palliative care professionals involved, weekly diaries filled out by consultants, and an implementation diary. Qualitative data were analyzed following the principles of thematic analysis. Quantitative data were analyzed descriptively.

**Results:**

Thirty-four consultations, 22 multidisciplinary meetings and 9 training sessions were studied during the implementation period of 21 months. Social service professionals made up the majority of all professionals reached by the intervention. In all regions the intervention was perceived to have added value for collaboration and networks of social service and palliative care professionals (connecting disciplines reciprocally and strengthening collaborations), the competences of especially social service professionals involved (competency in palliative care provision, feeling emotionally supported in complex situations), and the quality and timing of palliative care (more focus on quality of life and dying, advance care planning and looking ahead, and greater awareness of death and palliative care).

**Conclusions:**

The threefold consultation service particularly helps social service professionals connect with palliative care professionals. It helps them to identify palliative care needs in good time and to provide qualitatively better palliative care to persons experiencing homelessness.

## Background

Persons experiencing homelessness often live lives characterized by poor health, frequently in combination with psychiatric diseases, intellectual disabilities, behavioral issues, and/or substance use [1–3]. The official number of persons experiencing homelessness in the Netherlands—defined as persons living rough, in emergency accommodation, or in special accommodation for persons experiencing homelessness or housing instability—has risen from almost 18,000 in 2009 to almost 32,000 in 2021 [4, 5]. Persons experiencing homelessness often suffer from infectious diseases, cancer, psychiatric disorders, or cardiovascular disease [6–9] and have a higher risk of health problems as they grow older [1, 10]. If their health condition worsens, they often live in shelters for day care, night shelters, or respite care [11], which we refer to here as ‘social service facilities’. We term the professionals working at these services “social service professionals”; they include social-care workers and street nurses working on in-shelter nursing wards. In-shelter nursing wards are often small-scale nursing wards accommodated by social service facilities and providing in-patient nursing care. In this setting, homeless persons can be admitted for urgent somatic issues, for example, to recover after surgery or if they (unexpectedly) become bedridden and require nursing care. In social service facilities, somatic issues among persons experiencing homelessness are presented and regular deaths regularly occur. So-called street nurses provide this care, these are often generalist nurses who have a long history of working with this population [11, 12]. The age at death of the homeless population is approximately 10 to 30 years younger than for the housed population [13–19]. However, palliative care is often lacking, late or of poor quality [20–22]. Therefore, timely palliative care is of great importance. We define “timely palliative care” as recognizing the end of life in good time and early implementing palliative care practices. It is known that early palliative care is closely related to quality of life in the last phase of life [23, 24].

Several international studies and commentaries have shown a need to strengthen multidisciplinary collaboration between palliative care professionals and social service professionals in order to improve the access and quality of palliative care for persons experiencing homelessness [25–29]. We define palliative care professionals as nurses specialized in palliative care, elderly care physicians, and GPs with additional training in palliative care. A multidisciplinary approach to palliative care for persons experiencing homelessness is especially important due to the complex and multi-problem nature of their needs. In addition, the care in many shelters focuses on practical issues such as assistance by realizing stability in housing and work, and dealing with issues such as, for example, financial matters, substance use, or psychiatric diseases. There is less consideration for medical issues, illness, and death [20, 28]. Evidence on methods using multidisciplinary collaboration or staff training for professionals serving this population shows perceived positive effects on the quality of palliative care [30–32]. However, multidisciplinary collaboration between social service and palliative care professionals using a threefold intervention has not been studied yet within social service and healthcare professionals caring for persons experiencing homelessness.

In our previous focus-group study, we explored the need for a consultation service intervention in which professionals of different disciplines work together in order to increase collaboration and the exchange of knowledge between social service professionals and palliative care professionals [33]. Participants who experienced homelessness indicated that social service and healthcare professionals serving them need to be trained more in palliative care knowledge and skills, interdisciplinary collaboration, tailored care provision, and an attitude showing understanding and respect. They expected a consultation function supplemented by training and interdisciplinary collaboration to contribute to better palliative care for them.

In the Netherlands, palliative care is generally delivered by generalists who are supported by healthcare professionals specialized in palliative care. Consultation with a palliative care consultant is a commonly used method to increase palliative care knowledge among healthcare professionals, and it has been shown to lead to the delivery of palliative care starting earlier [34, 35]. Our focus-group study showed that reciprocal consultation between social service professionals and palliative care professionals was expected to be of added value in providing timely and appropriate care to persons experiencing homelessness who have palliative care needs. Additionally, we found that training and multidisciplinary meetings should be part of this approach as well. A context-sensitive development of this intervention was considered important in view of the differences between services and regions [33, 36].

Therefore, over a period of 21 months a threefold intervention involving consultation, multidisciplinary meetings, and training sessions was developed and implemented in three Dutch regions and the added value evaluated. The intervention was aimed at both professionals working in the field of social services for people experiencing homelessness and healthcare professionals working in the field of palliative care. We aimed to improve the quality of care by increasing collaboration and knowledge, and ensuring that palliative care starts earlier for people who are homeless.

In this evaluation study, we aim to provide insights into the intervention’s added value. The research questions were:

1. How was the threefold consultation service received by social service professionals and palliative care professionals, and which professionals and patients did the intervention reach?
2. What is the perceived added value of the threefold consultation service for collaboration between social service and palliative care professionals, professionals’ competences, and the timing and quality of palliative care for persons experiencing homelessness, according to the social service professionals and palliative care professionals?

## Methods

### Intervention

The intervention is described in Table 1.

**Table 1:** Description of the threefold consultation service

**Elements of the threefold consultation service**	
The intervention comprised three facets. These were: [1] regular reciprocal bedside consultations between social service professionals and palliative care professionals (seeing the patient and talking with them) concerning patients experiencing homelessness who are eligible for palliative care. Involved are professionals working in the field of social services for this population, and palliative care professionals working, for instance, in hospices and General Practices [2]; multidisciplinary meetings between social service professionals and palliative care professionals to discuss patients eligible for palliative care; and [3] training and education on both palliative care and homelessness, whereby the frequency and content were determined by the professionals concerned. Also ‘strategic partnerships’ were created with one consultant in palliative care and one consultant in services for the homeless. This pair of consultants formed the basis for the intervention; the consultants initiated the consultations, multidisciplinary meetings, training sessions, and the involvement of other organizations. Group meetings with all the consultants were scheduled every six months in each region. Social service facilities who participated in the intervention applied a recovery-approach, implicating that social service professionals stimulate and assist residents as much as possible to become as independent as possible. Within this recovery-approach, patient-centered care is a core element, focusing on the needs and wishes of the client.	
**Intervention duration**	
The implementation of the threefold consultation service and process evaluation started with a preparatory phase for all three regions from June to September 2019. Implementation plans were made, followed by an execution phase lasting 18 months. From March 2020, the COVID pandemic affected the evaluation. Professionals had less time and had additional tasks, while visiting restrictions meant interviews had to be conducted by phone or video call.	
**Context-sensitive approach and implementation plans**	
This intervention was designed to be context-sensitive in order to fit local needs and tie in with existing collaboration efforts and/or further develop them. The regions of Amsterdam, Rotterdam, and Utrecht (three large cities in the Netherlands) participated in this intervention. Part of the context-sensitive design involved working out strategies drawn up in implementation plans by the participating organizations in each of the three regions. These implementation plans covered: the details of organizing the consultations, existing initiatives for consultation, collaboration, knowledge exchange, training, the organization of multidisciplinary meetings and potential for improvement, the organization of training and additional educational requirements, barriers and facilitators for all three elements, characteristics specific to each region, and possibilities for future financing and the continuation and embedding of the intervention. The implementation plans were updated every six months on the initiative of the researchers.	
**Small-scale intervention**	
The intervention aimed to start on a small scale and to expand further amongst professionals in the region once the consultations, multidisciplinary meetings, and training were well established.	

### Design and data collection

The mixed-methods evaluation study consisted of structured questionnaires, semi-structured topic-list guided interviews, and diaries. Table 2 shows an overview of methods used for data collection. Participants were sampled purposively based on their participation in the intervention activities. They were then contacted by phone or via the consultant. Almost all interviews were audio-recorded and performed by phone by one researcher (HK) trained in qualitative research; there were also two audio-recorded face-to-face interviews. The 18-month evaluation started in September 2019 and ran simultaneously with the implementation.

**Table 2:** Overview of methods used, topics and content, respondents, and measurement moments

Methods	Topics and content	Respondents	Measurement moments
Structured questionnaires on consultations, including the perceived added value	Nature of care request, patient diagnosis, advice provided or received (broken down into the physical, psychological, social, and spiritual domains of palliative care, plus addiction), consultant’s knowledge, consultation timing, facilitating and impeding factors regarding consultation, consultation quality, concreteness and usefulness of advice, effect on quality of palliative care, and added value of consultations.	Consultant Professional who requested consultation Requesting consultant	After each consultation
Structured questionnaires about the multidisciplinary meetings	Professional background of attendees, diagnosis, and details of the patients and domains discussed.	Consultant	After each multidisciplinary meeting
Structured digital diary recording activities and experiences with these activities	Type and number of activities performed, reason for activity, experiences with activity. The activities were: consultations, multidisciplinary meetings, training (given or received), and project team meetings.	Consultant	Weekly
Semi-structured group interviews about the perceived added value of multidisciplinary meetings and training activities	Process of getting involved in multidisciplinary meetings and training activities, appreciation of collaboration and discussions, discussed topics, added value of meetings, effect on knowledge and competences, effect on timing and quality of palliative care, suggestions for improvement.	Attendees of multidisciplinary meetings and training activities	After 12 multidisciplinary meetings and training activities
Semi-structured individual interviews about activities, process, added value, and maintenance	Activities, process, added value, and maintenance.	Managers at participating organizations	Shortly after consultation
Semi-structured individual interviews about activities, implementation, and added value	Current activities, collaboration, implementation and effort required, useful elements, missing aspects, perceived benefits of the three elements, perceived added value for collaboration, competences, quality and timing of palliative care.	Consultant	Mid-intervention period
Implementation diary with observations on added value and factors affecting this	Observations on the intervention: activities performed, steps taken to accomplish this, and evaluations and difficulties in this process. Observations on implementation: support for this process, strategies.	Researchers	Every week

The data consisted of: 216 structured weekly digital diaries recorded by the consultants; 34 questionnaires filled out by consultants after consultations; 14 questionnaires completed by professionals who had requested a consultation; 22 questionnaires about multidisciplinary meetings (MDMs) filled out by the palliative care consultant; eight semi-structured individual interviews with managers at organizations involved in the intervention; two semi-structured group interviews about MDMs (*n* = 10, one face to face and one by video call); two semi-structured group interviews about the training (*n* = 10, one face to face and one by video call); five telephone interviews with consultants; and one implementation diary.

### Ethics

Participants were informed verbally by the researcher about the research goals. Written informed consent was provided by all the professionals involved in interviews prior to the interview. Completed questionnaires and transcripts were anonymized to ensure the participants’ anonymity. Access to the data was limited to three researchers. On July 24, 2019, the Ethics Review Committee of VU University Medical Center provided a waiver as ethical approval was not needed under Dutch law.

### Data analysis

Quantitative data were analyzed descriptively using SPSS 26.0 [37]. Answers to open questions were categorized by one researcher (HK) and checked by a second researcher (BO). Qualitative data collected in the interviews and the implementation diary were analyzed using the RE-AIM framework to structure analysis, and subsequently searching openly for themes within these RE-AIM dimensions, following the principles of thematic analysis [38]. RE-AIM is an acronym for the framework’s five evaluation dimensions: Reach, Effectiveness, Adoption, Implementation, and Maintenance [39]. Through these dimensions, the impact of innovations can be assessed. In this study, we focused on Effectiveness, described as perceived added value of the intervention. Reach is the extent to which the target population was reached by the initiatives. Adoption is the degree to which the initiatives were adopted or used by organizations and settings. Implementation is the extent to which the initiatives have been implemented according plan, including barriers and facilitators. Maintenance refers to the extent to which the initiatives are future-proof. The results on Reach, Adoption, Implementation and Maintenance were described elsewhere [40]. Using MAXQDA2020 [41], analysis started after conducting the first five interviews. After an initial analysis of these interviews, topic lists appeared to have some overlap, and the topic lists were shortened slightly. Three researchers (HK, BO, AV) independently coded four transcripts and then discussed their codes together until agreement was reached. Afterwards, all other data were coded by one researcher (HK). This researcher specifically looked for information on the reach of the intervention, collaboration between social service and palliative care professionals, social service and palliative care professionals’ competences, and the quality and timing of palliative care. In addition, an open and inductive search was made for new themes that emerged from the data, until no more new themes were found. All codes were grouped into themes, which were discussed in the research team until no more new themes appeared.

## Results

### The threefold consultation service during the intervention period

Table 3 shows the number of social service and palliative care professionals who took part and the activities within the three regions.

**Table 3:** Overview of social service and palliative care professionals and activities

	n of unique involved palliative care or healthcare professionals involved^**a**^	n of unique social service professionals^**b**^ involved	n of consultations	n of MDMs^**c**^	n of training/education sessions
Region 1	19	40	5	11	3
Region 2	18	19	5	7	3
Region 3	33	47	24	4	3
*Total N*	*60*	*106*	*34*	*22*	*9*

^a^Including nurses specialized in palliative care and hospice care, nurses specialized in psychiatric care, district nurses, home care nurses, GPs (in training), medical specialists concerning elderly care physicians (in training) and psychiatrists, spiritual caregiver, practice nurse, pharmacist

^b^Including care coordinators and managers of social service facilities, in-shelter nurses, social workers, residential workers, a secretary

^c^Multidisciplinary Meetings (MDMs). The meetings involved the individuals referred to in columns 1 and 2

In total, 60 palliative care professionals and 106 social service professionals were involved in the intervention. In the three regions combined, 34 consultations were held with social service professionals and palliative care professionals. All consultations were requested by social service professionals. A total of 22 multidisciplinary meetings were organized, as well as nine training sessions in the field of palliative care or in the field of social services for professionals serving people experiencing homelessness.

Table 4 shows that 54 patients were discussed in the consultations (*n* = 22) and multidisciplinary meetings (*n* = 32). Most of them were male (80%). Almost all patients (93%) were 70 years of age or younger. A majority of patients had Dutch nationality (59%) and resided in long-term shelters (57%). The most common diagnoses were cancer or lung disease (including COPD) and severe substance use.

**Table 4:** General characteristics of patients discussed in consultations and MDMs

	n of patients discussed in consultations (%)	n of patients discussed in MDM (%)	Total n (%)
Unique patients	22	32	54
*Region*			
Region 1	4 (18)	14 (44)	18 (33)
Region 2	4 (18)	15 (47)	19 (35)
Region 3	14 (64)	3 (9)	17 (32)
*Sex*			
Male	17^a^ (77)	26^b^ (82)	43 (80)
*Age range*			
30–40	0 (0)^a^	3 (9)	3 (6)
41–50	3 (14)	2 (6)	5 (9)
51–60	8 (36)	9 (28)	17 (32)
61–70	9 (41)	10 (32)	19 (35)
70 and older	2 (9)	2 (6)	4 (7)
*Nationality*			
Dutch	13^a^ (59)	n/a^e^	
European (non-Dutch)	1 (5)	n/a^e^	
Non-Western	8 (36)	n/a^e^	
*Residence*			
24-hour shelter (long term)	14 (64)^a^	17 (53)	31 (57)
Assisted living	3 (14)		3 (6)
In-shelter nursing ward	5 (22)	15 (47)	20 (37)
*Diagnoses*^*c, d*^			
Somatic	21^a^	26^b^	47 (87)
Cancer (metastatic)	9	15	24
Lung disease / COPD	4	5	9
Rheumatism	3	0	3
Heart failure	1	5	6
HIV	0	3	3
Other	4	5	9
Substance use	17	19	36 (67)
Tobacco	5	n/a^e^	5
Combination of substances	5	2	7
Cocaine and/or heroin	5	6	11
Alcohol	2	2	4
Methadone	1	n/a^e^	1
Unspecified	0	9	0
Mental health	12	4	16 (30)
Schizophrenia	6	0	6
Personality disorder	3	3	6
Other	3	1	4

^a^Missing values for consultation data were < 5% for sex, age, nationality, residence and diagnoses. Missing values are not included in the numbers and percentages

^b^Missing values for MDM data were 19% for age and 13% for diagnoses. Missing values are not included in the numbers and percentages

^c^As reported in the first consultation

^d^ ombination of diagnoses possible

^e^Not included as a question or a checkbox in the evaluation questionnaire after MDMs

Consultations between social service professionals and palliative care professionals lasted on average 56 minutes (range 10–120) and were mostly held at the bedside (59%). For practical reasons or due to COVID-19 restrictions, some consultations were by phone (32%). On average, 3.23 symptoms (range 1–6) were mentioned in a consultation request; these were mainly fatigue, pain, and weight loss. On average 1.79 domains (range 1–4) were covered in the consultation requests per patient. These requests mostly concerned somatic aspects (30/34) and the patient’s future well-being, pain treatment, or support and comfort in daily life. To a lesser extent, consultation requests concerned the psychological domain (7/34), for example patients being withdrawn or defensive. Some consultation requests were in the social domain (6/34), concerning the involvement of family members, isolation, or behavior. Lastly, consultation requests were in the existential domain (5/34), concerning future wishes, how to discuss incurability with the patient, isolation, and suffering. Consultants reported on average 2.23 (range 1–4) advice per consultation; these were mostly somatic care advice (29/34), and to a lesser extent advice on substance use (10/34), psychosocial aspects (19/34), and existential aspects (9/34).

On average MDMs lasted 75 minutes (range 30–90) and were attended by an average of 6.86 persons (range 4–9). In terms of disciplines, most attendees were general practitioners (GPs) and practicing or trainee medical specialists (34%), nurses and social workers in social services (21%) and to a lesser extent, in-shelter care coordinators (11%), nurses specialized in palliative care or psychiatric care (20%), or professionals of other disciplines (14%). The training sessions as part of the threefold consultation service lasted on average 132 minutes (range 30–300) and were attended by an average of 12.43 persons (range 7–16). Training sessions were provided on an introduction to palliative care, palliative sedation, handling complicated behavior, and taking care of caretakers.

### Added value of the threefold consultation service

The use of the intervention differed per region and strongly depended on what options for consultation, MDMs, and training were already available within the region and whether there were needs within the region. Table 3 shows use of the intervention elements by region. Although the three regions used elements of the intervention differently due to the tailored nature of the intervention, there was homogeneity in the perceived added value of the intervention.

The intervention mainly had perceived added value for collaboration between social service professionals and palliative care professionals and the competences of these professionals, which in turn was thought to improve the timing and quality of palliative care. The data revealed seven themes reflecting the added value of the intervention.

### Collaboration and professionals’ networks

#### Reciprocity helps connect social services and palliative care

The intervention resulted in reciprocity between healthcare professionals and social service professionals. For all intervention elements, reciprocity was of added value in equipping especially social service professionals as caregivers to provide palliative care in the physical, psychological, social, and spiritual domains to a complex population. Reciprocity provided these social service professionals with additional knowledge, skills, and understanding that helped them take care of this population. Social service professionals also gained awareness about options for palliative care provision that they had not been aware of before, which is illustrated in Table 5, Quote 1. Reciprocity resulted in particular in social service professionals having more palliative care competences Although palliative care professionals did not request consultations with social service professionals, reciprocity in the exchange of knowledge resulted in them having better insight into a complex population, and better knowing the needs of this population regarding, for example, addiction issues, as illustrated in Table 5, Quote 2.

**Table 5:** Quotes on collaboration, competences, and quality and timing of palliative care

Theme and subtheme	Quote number	Quote
Collaboration and networks		
*Reciprocity*	Q1	R: Well, she [palliative care consultant] was real good at tying in with the medical aspects, I’d say. So yes, she has a different view of the residents than we do as social service professionals. Yeah, we mainly focus on the supervisory aspect but we don’t know so much about the medical, physical aspect. She helps us think about that and asks us critical questions. She also does that with the psychiatrist and the family doctor. And she looks at what alternative solutions there are and how we can get an even better picture of a resident in terms of their physical condition. (Region 2, social service professional)
	Q2	But for me it’s the other way about, because of course I wasn’t familiar with the whole homeless world. I knew something through the addiction services because of the multidisciplinary meetings [MDMs, which the participant took part in before the intervention]. But I take things from that and I think that we... […] Well, that we got to know more and... well, that we learned from one another. [...] So not just more breadth, but also getting the nursing perspective more involved [...] (Region 1, palliative care consultant)
*Creating and strengthening collaborations*	Q3	I: Right, and what did you notice about the care providers’ experience with that? [familiar face and being able to consult someone] R: Yeah, they like that. Well, she’s much more accessible, you can easily just phone her, she’s in the MDM so you can raise things there. Right, of course that’s really nice. Palliative care is always a bit of an issue... I: Is it? R: Yes, because someone’s dying and that’s always a bit... well, some people have trouble with that. With morphine too, you know, giving morphine. Imagine we gave him morphine and then he died, did I... is that the reason he died? That’s another issue. So it’s always nice if you have someone you can fall back on then. (Region 1, Manager 4)
	Q4	I think that we can find one another, and there comes a point that we’re in one another’s networks. And if we had a patient here with really complex behavior, then even if they came from another homeless center and not from [the homeless center with nursing facilities], for example, then you can still just call [the homeless center with nursing facilities] and ask them what we ought to do, or what route we need to take to get help with this. (Region 1, palliative care consultant)
Competences of professionals involved		
*Feeling equipped and competent in palliative care provision*	Q5	R: For example by agreeing conduct-related things with your co-workers in training sessions or peer review sessions, and by clearly demarcating the clinical picture. Right: why do you still want to send that man to hospital and what are you hoping to achieve? You’re fighting a losing battle. And you see that you can help the team make decisions about such things. (Region 2, Manager 2)
	Q6	[Palliative care consultant] sees and hears all the things that are going on, naturally enough, because she works here [in the hospice] and at [social service facility] so she sees what people are up to and what issues they face. Sure, [palliative care consultant] focuses her advice on palliative care, but she also learns a lot about the problems, the needs and especially the behavioral issues of homeless people. That experience is something she takes with her, consciously or subconsciously, in her work here [the hospice]. (Region 1, Manager 3)
*Support in complex and emotional situations*	Q7	R: I know she was involved at [location 2] with someone who was really in the terminal stage... and also behaviorally... I’m not going to the nursing home. And she was really good there at helping think up solutions, whereas the family doctor said, ‘Just go to a nursing home.’ And we thought, that’s not always how it works. It’s different behavior, another culture, not wanting to leave your safe environment, not having any family. She gave real support — what can you do as a team? [...] She could act as an intermediary more, say this is what the team can do, these are their areas of expertise, but that only goes so far and after that, we need to bring in other people. She brings those different worlds together. You really need that with a specific group, especially here with aging people and addiction and Lord alone knows what diseases. Yes, the whole package is broader, more complex perhaps. She’s better at that. I don’t have a nursing background at all. But the ultimate responsibility for the processes is mine, so I thought, ‘Oh great, someone who’s helping find solutions, that can only be positive.’ (Region 3, social service professionals in MDM)
	Q8	R: A nice added advantage is that you’re then obviously helping increase the knowledge of the person who requested the consultation. One aspect of the consultation is often that you say, wow; that you show to a certain extent how it’s tough and difficult for the person requesting the consultation too. So that’s directly for the client and indirectly for the consultation requester. [...] But the training focuses mainly on the care providers... and they’ve been left out of the picture for a very long time. (Region 2, palliative care consultant)
Quality and timing of palliative care		
*Focus on quality of life and dying*	Q9	R: She is also doing a bit better now thanks to this [dietary advice]. You can have a bit more of a conversation with her, and she’s slightly more cheerful and less at risk of falls. So at first it’s like, yeah, she’s getting so thin, we can’t communicate with her, everything’s getting worse, one function after another stops working. Not caring properly for herself. And now you see her becoming more stable purely thanks to a good diet. And that it could be a few years yet. But then you prioritize... you prioritize comfort rather than active treatment. Then you can say it’s a palliative process because if this woman doesn’t want to go any further we need to accept it, or if she starts to feel a bit better again we could raise the topic again. That’s the great thing about this; they arranged an Italian interpreter and now she has agreed to an operation, a cataract operation. Well, that’ll also improve her quality of life because she’ll be able to see things a lot better then, she will be able to watch TV, all that kind of thing. Those are things she enjoys doing. (Region 3, palliative care consultant)
	Q10	R: Um, well, it [the advice in consultations and the MDMs] gives you a better understanding anyway if you know someone’s in the final stage. Including stuff about how you can keep up their quality of life. Um, also the fact that you can discuss it with the actual resident. Just like when someone has poor liver function and you can tell them: look, if you carry on like this, it will eventually be too late. And your liver is functioning really badly. R: It’s terminal... R: Yes. (Region 3, MDM)
*Advance care planning and looking ahead*	Q11	Well, I think the quality of the palliative care in [social service facility] is improving as a result. They’ve gotten better at preparing for the things that could happen. So the advance care planning is better, so they don’t end up facing a *fait accompli*, a problem where they’re saying, “Help, what do we do now?” But it becomes, well … so that they have a better idea of what they can expect and therefore be better prepared and are better able to anticipate wishes, so the clients also get better care. (Region 1, Manager 2)
	Q12	R1: But then at one point she [palliative care consultant] asked a kind of question: think of your clients — if they were to die within a year, would you be surprised or not? I learned from that... going from intuition to action where medical matters are concerned... that we should run through those clients and ask ourselves, why are you actually so concerned about them? ...And she [palliative care consultant] knew that you could use this here, that study, or try that. R2: Right. So there are more options. R1: That’s my main experience. I genuinely had to learn that difference between palliative and terminal. I didn’t know about that. R2: A lot of people didn’t know about that. It really has been a lesson. (Region 3, MDM)
*Awareness of death and palliative care*	Q13	R: Well, then I’m also a personal mentor [of clients in social services], and she [palliative care consultant] asked for medical data, and what’s the situation with the phase of life, what stage are we in? And if it’s a real bad stage, well then you know that at some point you can start a palliative route, that it’s possible that person could die soon. That was a really new perspective for us because we didn’t know much at all about that aspect. Right, we don’t know when... you don’t know that anyway, but the fact that you also look at the physical aspect, at how far the resident has gone there, that was genuinely new, let’s say. I: Right, so a world kind of opens up showing what you can learn about this and what can be going on physically? R: What can be going on physically and how far that may have progressed. (Region 3, MDM)
	Q14	R: Right, but people [social service professionals] do need to be open to this and that’s not always the case. I: Right, so you can also run into quite a lot of...? R: Resistance. Yes, simply resistance in the sense of that’s not part of our job, we don’t do that. But when that occurs, you just let it happen. So yes, it’s a bit of give and take. [...] And you know what, homeless people, or the people in our target group, usually don’t have any family with them anymore. So if something happens and someone dies suddenly or didn’t get as much care as they needed, well, there’s no one to sound the alarm, let’s say. That’s not something the residential care facility does deliberately, but it’s that knowledge that’s lacking again. Sometimes I think there’s not quite enough attention for the real terminal care for such clients. (Region 3, Manager 1)
	Q15	[Palliative care consultant] notes that there is a lot of uncertainty and a need for palliative care advice or simply to see whether someone is deteriorating. Her team meetings and recommendations are mainly about comfort and quality of life. She sees a lot of unconscious incompetence among care providers at the residential locations. Now sheltered housing supervisors are asking her for advice if they are worried about a resident or if they think someone will die if things continue like this. (Region 3, implementation log book)

#### The intervention creates and strengthens networks and collaborations

Mainly because of the MDMs and training sessions, the intervention resulted in new formal networks and new forms of teamwork encompassing professionals in social services and palliative care. The main added value of these networks and this teamwork was in knowing how to find one another, the ‘strategic partnerships’ (Table 1), and familiarity, which makes it easy to consult one another. Quote 3 in Table 5 illustrates this. Other perceived benefits were the new options for patient transfers, contacts with new categories of professionals such as spiritual caregivers, and being able to integrate the two ‘worlds’ of healthcare and social services, as expressed in Quote 4 in Table 5. On the other hand, some social service professionals said they felt no need for more collaboration as they already felt committed to in-house palliative care for their patients due to the involvement of a GP. Also, some social service and palliative care professionals reported that they were not yet able to assess the added value of the intervention due to a delayed start, the small number of activities, or only briefly being engaged as a consultant in the intervention (Table 5, Q2).

### Social service and palliative care professionals’ competences

#### Professionals feel better equipped and more competent in palliative care provision to this specific population

Both social service professionals and palliative care professionals felt better supported in providing palliative care to patients coping with complex issues in all domains of palliative care. Advice in consultations and MDMs on medication, and symptom management provided in consultations by palliative care professionals helped social service professionals feel more competent in detailed, early and comfortable palliative care provision. Training helped them feel better equipped in applying knowledge, guidelines, and protocols. These tools, knowledge and skills were applied to signal deterioration of the patient and decide about subsequent policy and actions and to monitor the resident. An example of recognizing deterioration and whether or not to send someone to the hospital for further treatment is exemplified in Quote 5, Table 5. Trained social service professionals felt that they could bring in ‘fresh’ expertise within their team, that they were better able to consider the situation from multiple perspectives, had easier access to palliative care services, and could request a consultation more easily. These benefits of the intervention were experienced in particular by social service professionals whose organizations did not have palliative care expertise or who previously felt ill equipped to provide palliative care to this patient population In addition, trained palliative care professionals gained knowledge about how to show attitudes reflecting understanding of the needs of this population, and about how to limit one’s unrestricted behavior, which is illustrated in Quote 6, Table 5.

#### Social service professionals feel supported in complex and emotional situations

Being able to request a consultation or ask for a second opinion on suspicions or actions that had been taken was perceived as of great importance for the professionals involved in the intervention. Social service professionals in particular reported that being able to consult a palliative care specialist whom they already knew helped them feel emotionally supported, gave them self-confidence and let them provide tailored care to the patient. Another perceived benefit was self-efficacy due to feeling better equipped, feeling supported when making decisions, and being able to ask for help, which we illustrate in Quote 7 (Table 5). Quote 8 (Table 5) show that palliative care professionals also feel the urge of emotional support of social service professionals. Some consultants, however, had ambiguous feelings about the added value of the intervention as it increased their workload and caused stress as well.

### Quality and timing of palliative care

#### More focus on quality of life and quality of dying perceived by both social service and palliative care professionals

Social service professionals and palliative care professionals saw added value for the quality of life and quality of death of patients from the regular use of all three elements of the intervention. They felt that the involvement of a palliative care consultant, the knowledge gained or refreshed in training, and the discussions of patients in MDMs with other professionals helped social service professionals offer residents more comfort and prioritizing comfort when someone is detoriating, which is illustrated by Quote 9, Table 5. Also, better symptom management and monitoring, and more humanity, with a greater focus on the residents’ needs and wishes, and on somatic aspects of palliative care for residents residing in social service facilities. These factors, related to better understanding, recognizing and discussing palliative care and the specific needs of persons experiencing homelessness were perceived to contribute to quality of life, as illustrated by Quote 10 (Table 5).

#### Focus in social services on palliative care encourages advance care planning and looking ahead

In line with the previous theme, the training sessions and consultations in particular helped social service professionals use advance care planning for all their residents, as many of them are vulnerable, and look ahead. Quote 11 (Table 5) illustrates the use of advance care planning and the benefits thereof. Besides this, as many deaths seemed to occur suddenly, social service professionals perceived added value in spending effort on looking ahead, thinking about what could be expected in the future and how potential palliative care needs could be identified. The consultant’s questions during MDMs or consultations made them feel more alert to the possible deterioration in patients and made them more aware of early, tailored palliative care. Quote 12 (Table 5) illustrates that by using the surprise question, social service providers became alert in recognizing life-threatening illness, expressing and analyzing their concerns about specific residents, and starting concrete actions.

#### Awareness of death and palliative care among social service professionals increases

As a recovery-oriented and person-centered approach was shown to be common among social service professionals who participated in this study, they often lack knowledge and awareness on the possibility of palliative care and especially attention for the somatic domain (illustrated by Quote 13, Table 5). Social service professionals often felt too busy with day-to-day ad hoc issues and focused on regaining stability in the residents’ lives, and felt little or no concern with whether a resident could deteriorate. In addition, it was difficult for them to recognize whether a resident’s health was worsening. Table 5, Quote 14 illustrates the little attention that palliative care sometimes receives within the social service facilities. Because they often saw the resident on a daily basis, minor deteriorations in health went undetected. Training sessions, MDMs, and consultations made them realize that life is finite, and what consequences this has for daily care, as explicated in Quote 15 (Table 5).

## Discussion

All three elements of the threefold consultation service were used by all three regions, although regional differences in the way of use were found. Consultations mainly involved palliative care professionals advising social service professionals. Social service professionals mainly posed questions about somatic issues, whereas the provided advice often covered somatic as well as psychosocial aspects. In all regions social service professionals and palliative care professionals perceived an added value of the intervention on collaboration, competences, and quality and timing of palliative care.

Our findings reveal that the intervention is of perceived added value regarding the detection of possible palliative care needs and quality of palliative care. All three elements of the three-faceted intervention contributed to the perceived added value of the intervention as a whole, combining equipping social service professionals with knowledge, raising awareness, and facilitating collaboration. In terms of improving the quality of palliative care, our study revealed several aspects that have been identified as determining quality in palliative care by the Dutch Quality Framework for palliative care, namely recognition, proactive care planning, coordination and continuity, expertise, and personal balance of the professional [24]. Therefore, we consider this intervention as contributing to an improvement in palliative care for this population, whereby the training, MDMs, and consultations increase social service and palliative care professionals’ (conscious) competence.

Our study shows that collaboration between different organizations helps social services to deliver palliative care covering the somatic, spiritual, social, and psychological domains as defined by the WHO [23], rather than mainly from the social perspective as social services are used to doing. Several studies confirm the added value of interventions aimed at training or interdisciplinary collaboration between social service and palliative care professionals [30, 31, 42], resulting in more knowledge, skills, collaboration, and confidence when providing palliative care to this population. Other international studies confirm the need for palliative care in the place where persons experiencing homelessness reside, all the more because of this population’s poor access to palliative care [20, 21, 43, 44].

In addition to our findings on collaboration, timing and quality, and competences, our study highlights the emotional support needed by especially social service professionals when providing palliative care to this population. This is also found in other studies [44–47], which indicate a need for emotional support among social service professionals in demanding situations, such as the imminent death, extreme suffering or sudden death of patients with whom they have established a bond during the patient’s stay in the social service facility. Our study demonstrates the added value of the palliative care consultant in providing emotional support to social service professionals.

The diversity in use of the intervention but comparable perceived added value confirms the importance of a context-sensitive approach. Moreover, a context-specific approach per region using the basic elements of collaboration. Competences, and quality and timing of palliative care is relevant in other regions and countries. However, the success of implementing an intervention is also highly dependent on the process of implementation. Implementation of the intervention took time and effort, and some social service and palliative care professionals involved said that it was too early to assess the efficacy of the intervention or that more time was needed for better implementation. The COVID-19 pandemic made it particularly difficult to build new collaborations. A process evaluation is needed to gain more insight.

### Strengths and limitations

An important strength of this study is that it evaluates a new, regionally tailored intervention aimed at improving the quality and timeliness of palliative care for persons experiencing homelessness. Another strength of this study is the intervention explored and designed in close collaboration with persons experiencing homelessness, guided by our previous focus group study in which persons experiencing homelessness indicated that both social service and healthcare professionals serving them needed more knowledge, training and collaboration when providing palliative care [33]. The design of this intervention and the project were supervised by an expert by experience who participated in the advisory board. Also, in this study professionals were involved from both social services and health services, including palliative care professionals and their managers.

A limitation of this study is that persons experiencing homelessness who were the subject of consultations were not interviewed themselves due to COVID-19 visiting restrictions, although this had been intended in the study protocol. We recommend to conduct research into evaluation of this intervention from the perspective of those who experienced the intervention while measuring patient-centered outcomes. Another limitation can be seen in the use of the intervention within social services, which might have resulted in a relatively old, mainly Dutch patient population receiving care. Our study population is probably not generalizable to the entire homeless population in need of palliative care in the Netherlands, because a small number of seriously ill people live on the streets, are undocumented or do not use social services [4, 5].

## Conclusions

A threefold consultation service can help especially professionals in social services to connect with palliative care professionals. It is perceived to help professionals in social services better identify palliative care needs, and collaboratively provide timely palliative care of better quality. It is recommended to further study future use of the intervention in other regions and countries.

## Data Availability

The datasets generated and/or analyzed during the study are not publicly available due to the small scale of this intervention and evaluation and the easily traceable nature of de data, but are available from the corresponding author on reasonable request.

## Abbreviations

GP: General Practitioner
MDMs: Multidisciplinary Meetings
WHO: World Health Organization
